# Prognostic and immune-related value of complement C1Q (C1QA, C1QB, and C1QC) in skin cutaneous melanoma

**DOI:** 10.3389/fgene.2022.940306

**Published:** 2022-08-30

**Authors:** Huanglong Yang, Dehui Che, Yuxiang Gu, Dongsheng Cao

**Affiliations:** Department of Plastic Surgery, The Second Affiliated Hospital of Anhui Medical University, Hefei, China

**Keywords:** complement C1Q, cutaneous melanoma, prediction, prognosis, immuno-therapy

## Abstract

**Background:** Skin cutaneous melanoma (SKCM) is a common malignancy that is associated with increased morbidity and mortality. Complement C1Q is composed of C1QA, C1QB, and C1QC and is involved in the occurrence and development of many malignant tumours. However, the effect of C1QA, C1QB, and C1QC expression on tumour immunity and prognosis of cutaneous melanoma remains unclear.

**Methods:** First, we analysed C1QA, C1QB, and C1QC expression levels and prognostic values using Gene Expression Profiling Interactive Analysis (GEPIA) and Tumour Immune Estimation Resource (TIMER) analysis, and further validation was performed using RT-qPCR, The Human Protein Atlas, The Cancer Genome Atlas (TCGA) dataset, and Gene Expression Omnibus dataset. We then performed univariate/multivariate Cox proportional hazard model, clinicopathological correlation, and receiver operating characteristic curve analysis using TCGA dataset and established a nomogram model. Differentially expressed genes associated with C1QA, C1QB, and C1QC in SKCM were identified and analysed using LinkedOmics, TIMER, the Search Tool for the Retrieval of Interacting Genes database, and Metascape and Cytoscape software platforms. We used TIMER, GEPIA, and single-sample gene set enrichment analysis (ssGSEA) to analyse the relationship between the three genes and the level of immune cell infiltration, biomarkers, and checkpoint expression in SKCM. Finally, GSEA was utilized to study the functional pathways of C1QA, C1QB, and C1QC enrichment in SKCM.

**Results:** The overexpression of C1QA, C1QB, and C1QC provided significant value in the diagnosis of SKCM and has been associated with better overall survival (OS). Multivariate Cox regression analysis indicated that C1QA, C1QB, and C1QC are independent prognostic biomarkers for patients with SKCM. Immune cell infiltration, biomarkers, and checkpoints were positively correlated with the expression of C1QA, C1QB, and C1QC. Furthermore, the results of functional and pathway enrichment analysis showed that immune-related and apoptotic pathways were significantly enriched in the high-expression group of C1QA, C1QB, and C1QC.

**Conclusion:** We found that C1QA, C1QB, and C1QC can be used as biomarkers for the diagnosis and prognosis of SKCM patients. The upregulated expression levels of these three complement components benefit patients from OS and may increase the effect of immunotherapy. This result may be due to the dual effects of anti-tumour immunity and apoptosis.

## Introduction

Melanoma is a highly malignant tumour that originates from melanocytes (Kozovska et al., 2016). Melanocytes are found in the skin, eyes, digestive tract, and reproductive system; however, skin melanomas, which are caused by cancerous changes in melanocytes, are the most common type of melanomas (Dominiak et al., 2016; Kuk et al., 2016). Over the past few decades, the incidence of melanoma has rapidly increased (Rastrelli et al., 2014; Berwick et al., 2016). Certain factors may promote the malignant transformation of melanocytes, such as UV exposure from the sun, white race, trauma, or adverse stimuli (Bloethner et al., 2009; Darmawan et al., 2019). Melanoma is the deadliest skin tumour with characteristics of high malignancy, early metastasis, high mortality, and poor prognosis (Caini et al., 2015; Sun et al., 2019). Therefore, there is an urgent need for new insights into the molecular mechanisms that lead to the occurrence and progression of skin cutaneous melanoma (SKCM) and to promote the development of biomarker targets and novel therapeutic options to improve the survival rate of patients.

The complement system is a key component of both adaptive and innate immune systems (Laskowski et al., 2016). C1Q can perform various immune and non-immune tasks in a complement-dependent or complement-independent manner (Kishore et al., 2016). C1Q is a recognition molecule in the classical complement pathway and is well known as a subunit of the first complement structure, C1 (Espericueta et al., 2020). It is involved in a series of pathological and physiological activities, including the occurrence and progression of cancer, elimination of immune complexes, infection of the body, and apoptosis of cells (Kishore et al., 2004; Yang et al., 2019). C1Q is overexpressed in multiple types of cancer microenvironments (Bulla et al., 2016), and its potential anti-tumour effects have been reported in several studies. Kaur et al. (2016) found that C1Q can upregulate the TNF-α-induced apoptosis pathway by regulating Bax and Fas, thereby promoting the apoptosis of ovarian cancer cells. Hong et al. (2009) reported that C1Q can promote apoptosis of prostate cancer cells by stimulating the tumour suppressor WOX1. In a study by Bandini et al. (2016), it was reported that C1Q prevents tumour angiogenesis and induces apoptosis in breast cancer cells by coordinating WOX1-related signalling pathways. However, C1Q has been shown to increase primary cell adhesion, migration, and proliferation of malignant pleural mesothelioma (Agostinis et al., 2017). This contradicts the anti-tumour effect of C1Q mentioned previously (Mangogna et al., 2019a; Mangogna et al., 2019b). Therefore, the specific role of C1Q in cutaneous melanoma requires further investigation.

C1Q consists of 18 polypeptide chains, including six C1QA, six C1QB, and six C1QC (Nayak et al., 2012). Recent studies have confirmed that C1QB is a reliable biomarker for identifying patients with early cutaneous melanoma and observing treatment outcomes in follow-up patients (Luo et al., 2011; Borden et al., 2021). Although these results are encouraging, the tumour immune infiltration relationship between C1QA, C1QB, C1QC, and SKCM has not been established, and the expression, mechanism of action, and prognostic significance of complement components in SKCM require further exploration. In the present study, we found that these three genes were highly expressed in SKCM and have been verified experimentally. The relationship between prognosis and clinical features of melanoma was analysed, and a nomogram map was created based on clinicopathological features. In addition, hub genes related to the three genes were analysed. We then explored the relationship between the expression of the three genes and tumour immunity in SKCM. Finally, GSEA was performed to understand the pathways of action of the three genes. Our findings will help reveal the multifaceted roles of C1QA, C1QB, and C1QC and provide evidence for future immunotherapy of melanoma.

## Materials and methods

### Data source

**FIGURE 1:**
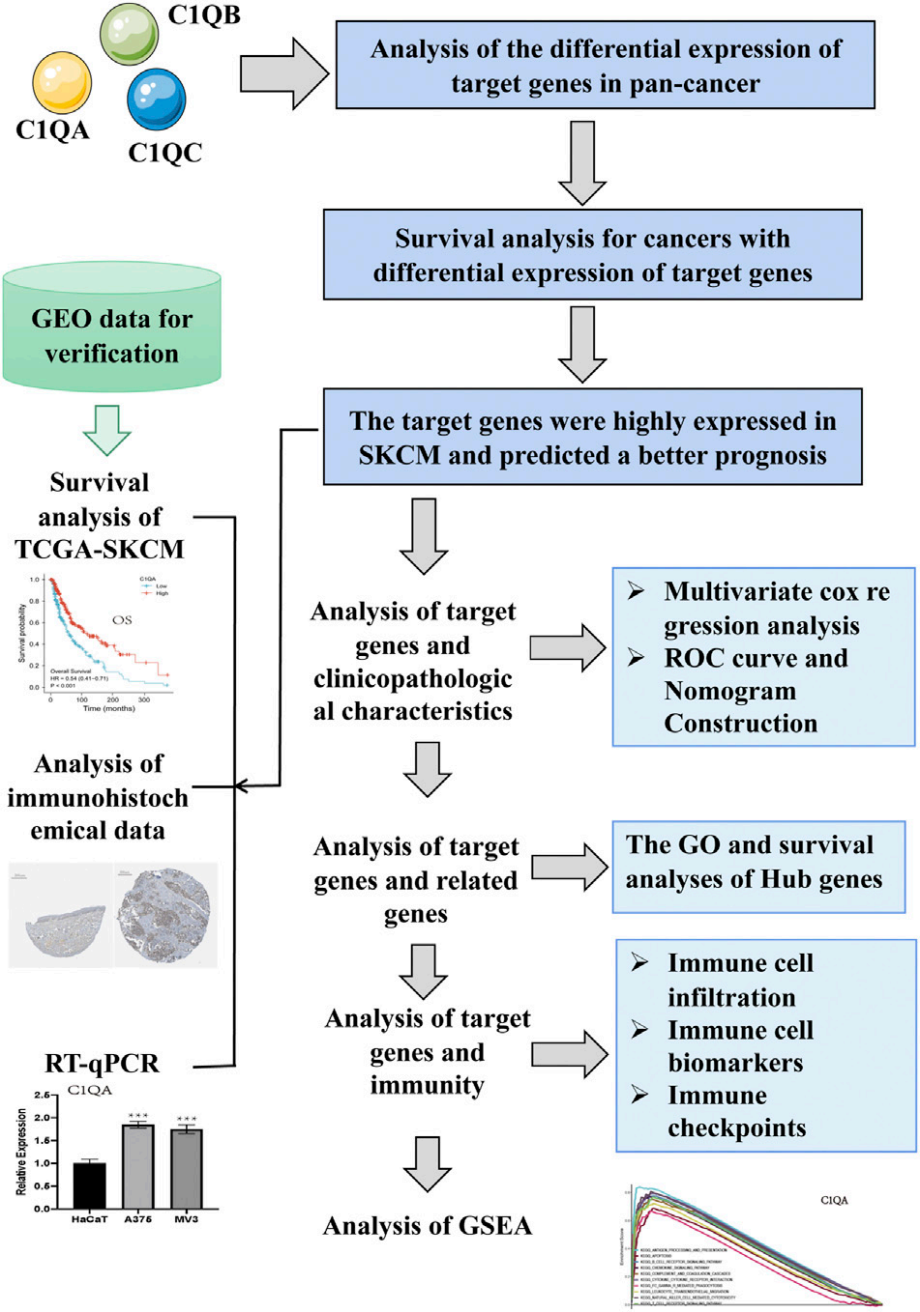
Schematic representation of the study workflow.

RNA transcriptome data (including 472 samples) and corresponding clinical profile data for clinicopathological stage, age, sex, and survival time for SKCM were obtained from The Cancer Genome Atlas website (TCGA, https://portal.gdc.cancer.gov/). Microarray data and clinical survival data for the validation cohorts GSE65904 (including 214 samples) and GSE53118 (including 79 samples) were downloaded from the Gene Expression Omnibus database (GEO, https://www.ncbi.nlm.nih.gov/geo/). We then eliminated samples with insufficient or missing information for further analysis. The research process is illustrated in Figure 1.

### Gene Expression Profiling Interactive Analysis and Tumour Immune Estimation Resource analysis

GEPIA (http://gepia.cancer-pku.cn/index.html) is an analysis tool developed based on TCGA and Genotype-Tissue Expression databases, which contain a large amount of RNA sequencing data and provide users with powerful functions such as differential expression analysis, correlation analysis, similar gene detection, and dimensionality reduction analysis (Tang et al., 2017). The TIMER (https://cistrome.shinyapps.io/timer) database contains more than 10,000 cases of 32 cancer types, which are used to estimate the level of six tumour infiltration immune subtypes and analyse tumour–immune interactions, including the relationship between immune infiltration and clinical characteristics, somatic mutations, and gene expression (Li et al., 2017). This provides a rich resource for cancer research.

In the current study, we used the GEPIA database to analyse the expression levels of C1QA, C1QB, and C1QC in normal samples and pan-cancer samples and screened out tumour types with significant expression differences for the subsequent analysis. Based on the analysis results of GEPIA and specific analysis modules in the TIMER database, we evaluated the correlation between the expression of C1QA, C1QB, and C1QC with overall survival (OS), abundance of immune cell infiltration, and immune checkpoint in SKCM patients and provided a reference for the selection of potential immunotherapy drugs. Finally, we validated the expression of proteins encoded by C1QA, C1QB, and C1QC in SKCM using immunohistochemical data from The Human Protein Atlas (HPA; www.proteinatlas.org) database (Uhlen et al., 2015).

### Assessment of diagnostic ability and construction of a nomogram

The receiver operating characteristic (ROC) curve can be used to test the reliability of C1QA, C1QB, and C1QC in the diagnosis of SKCM. The larger the area under the ROC curve, the stronger the diagnostic capability. The nomogram survival prediction model is based on a multiple regression model that visualises genetic and clinical features on a scale to predict 1-, 3-, and 5-year survival probabilities. We then evaluated the prediction accuracy of the nomogram using the compliance index (C-index) and calibration curve and visualised the results using “pROC,” “ggplot2,” “survival,” and “rms” R packages.

### Related gene analysis

Differentially expressed genes (DEGs) that were closely related to C1QA, C1QB, and C1QC were screened using the LinkedOmics (http://www.linkedomics.org) website. Then, the protein–protein interaction (PPI) network analysis was performed on C1Q-related genes with a correlation >0.7 using the Search Tool for the Retrieval of Interacting Genes database (STRING, https://string-db.org/) (Szklarczyk et al., 2021). Subsequently, based on the aforementioned PPI analysis results, Cytoscape software and cytohubba plug-in were used to screen the ten genes with the largest number of adjacent network nodes of C1Q-related genes as hub genes. Finally, the Metascape (Metascape) and TIMER websites were used to perform Gene Ontology (GO) enrichment analysis and survival analysis on these hub genes.

### Single-sample gene set enrichment analysis

ssGSEA could quantify the relationship between C1QA, C1QB, and C1QC in TCGA dataset and the infiltration of 24 types of immune cells (Bindea et al., 2013). Also, the “GSVA” R package was used to visualise the correlation between these three genes and immune cells, as well as the level of immune cell infiltration in the high- and low-expression groups of genes (Hanzelmann et al., 2013). The size of the circles and height of the bars represent the degree of correlation, and the shade of colour represents the magnitude of the *p-*value.

### Gene set enrichment analysis

Based on GSEA, an application program that evaluates the distribution trend of pre-defined genes in the gene table of two states and their contributions in two biological states, we analysed the enrichment pathways of C1QA, C1QB, and C1QC in the high- and low-expression groups. Each stage of the analysis was repeated thousand times to ensure the accuracy of the results.

### Culture of cell lines

SKCM cell lines A375 and MV3 and normal human HaCaT cells were obtained from the American Type Culture Collection (ATCC, Rockville, MD, United States) and Cell Lines Service (CLS, Eppelheim, Germany), respectively. A375 and HaCaT cell lines were cultured in DMEM (Shanghai Basalmedia Technologies Co., Ltd., Shanghai, China), while the MV3 cell line was cultured in RPMI-1640 medium (Shanghai Basalmedia Technologies, Shanghai, China). All media were supplemented with 10% foetal bovine serum (FBS; Gibco, Carlsbad, CA, United States) and penicillin (100 IU/ml)/streptomycin (100 μg/ml). The parameters of the humidified incubator were set to 37°C and 5% CO_2_.

### Reverse transcription-quantitative PCR

After extracting the total RNA of cells using conventional methods, the HyperScriptTM III RT SuperMix for qPCR with gDNA Remover (NovaBio, Shanghai, China) was used to convert it into cDNA. Subsequently, real-time PCR was performed using the S6 Universal SYBR qPCR mix (NovaBio) on a Bio-Rad iCycler Real-time Quantitative PCR system (Bio-Rad, CA, United States). Relevant primer sequences are listed in Table 1. The relative expression levels of the target genes were normalised to those of GAPDH and analysed using the 2^−ΔΔCt^ method.

**TABLE 1:** PCR primers utilized in this study.

gene	sequence (5ʹ to 3ʹ)
C1QA-F	CCA​TAT​CGC​TGG​CCT​CTA​TGG
C1QA-R	GTC​TTC​CTG​CCT​CCC​CTT​TC
C1QB-F	GGC​CAC​CGA​CAA​GAA​CTC​ACT​AC
C1QB-R	CCA​TAT​CTG​GAA​AGA​GCA​GGA​ACC
C1QC-F	AAC​CAA​TCA​GGT​CAA​CTC​GGG
C1QC-R	CCC​ACC​ATG​TCG​TAG​TAG​TCA​TTG
GAPDH-F	AAG​GTG​AAG​GTC​GGA​GTC​AAC
GAPDH-R	GGG​GTC​ATT​GAT​GGC​AAC​AAT​A

### Statistical analysis

R (v 3.6.3) software (https://www.r-project.org/) was used for data processing and statistical analysis of TCGA-SKCM and GEO cohorts, and *p* < 0.05 was considered to be statistically significant. Then, the “survival” and “survminer” R packages as well as the log-rank test were used to show the Kaplan–Meier plots. The “survival” R package was used to perform univariate/multivariate Cox risk regressions of three genes’ expression and clinicopathological features. The “ggplot2” R package was used to investigate the relationship between three genes’ expression and clinicopathological characteristics.

## Results

### mRNA expression levels of C1QA, C1QB, and C1QC in pan-cancer

**FIGURE 2:**
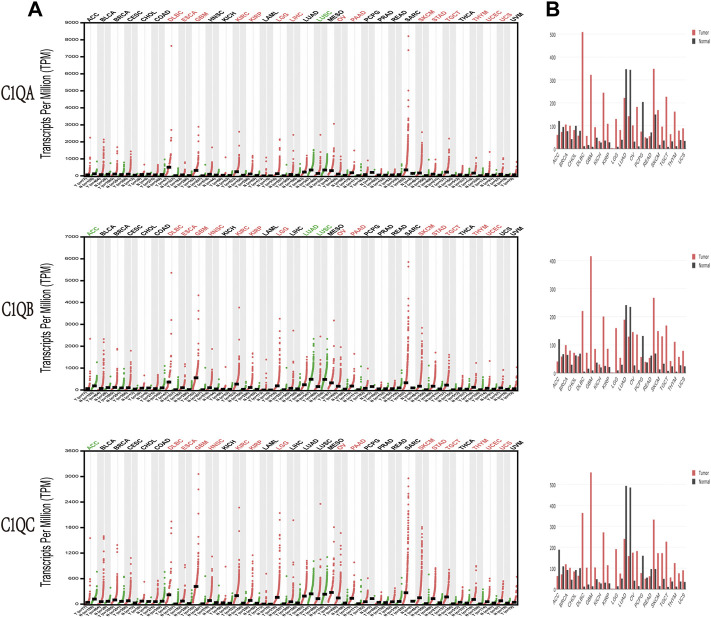
Expression levels of C1QA, C1QB, and C1QC in different types of human cancers compared with paired normal tissues by Gene Expression Profiling Interactive Analysis. **(A)** Dot plot. **(B)** Bar plot.

The transcription levels of C1QA, C1QB, and C1QC were analysed based on pan-cancer data in GEPIA to observe whether there were significant differences in their expression among common tumour types. As indicated in Figure 2, compared to normal tissues, C1QA was significantly upregulated in 15 types of cancer, namely, DLBC, ESCA, GBM, KIRC, KIRP, LGG, LIHC, OV, PAAD, SKCM, STAD, TGCT, THYM, UCEC, and UCS, while it was downregulated in LUSC. In addition, the expression of C1QB was considerably increased in 14 types of malignancies, namely, DLBC, ESCA, GBM, HNSC, KIRC, KIRP, LGG, OV, PAAD, SKCM, STAD, TGCT, THYM, and UCEC but dramatically decreased in ACC, LUAD, and LUSC. Furthermore, C1QC was significantly upregulated in DLBC, ESCA, GBM, HNSC, KIRC, KIRP, LGG, OV, PAAD, SKCM, STAD, TGCT, THYM, UCEC, and UCS but significantly downregulated in ACC.

### C1QA, C1QB, and C1Q were highly expressed in skin cutaneous melanoma and predicted a better prognosis

**FIGURE 3:**
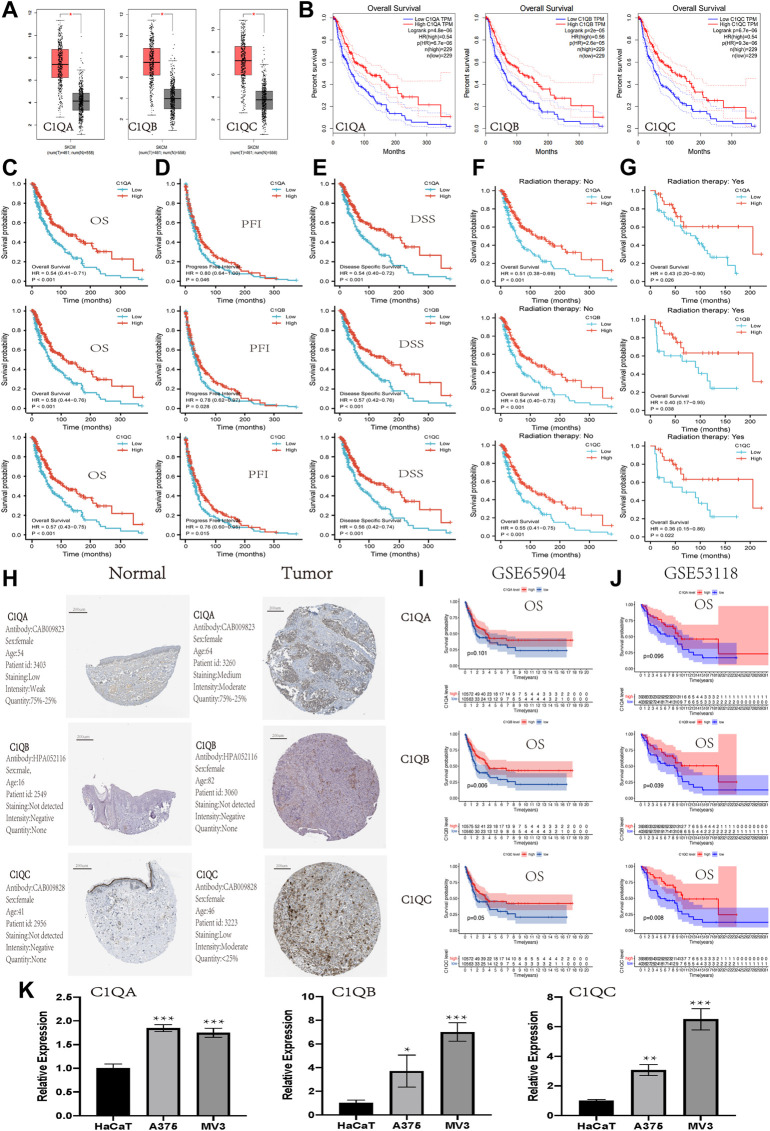
Expression and prognostic value of C1QA, C1QB, and C1QC in SKCM. **(A-B)** Expression and prognostic significance of C1QA, C1QB, and C1QC in SKCM analysed using GEPIA. The prognostic significance of C1QA, C1QB, and C1QC in SKCM from TCGA: **(C)** overall survival; **(D)** progress-free interval; **(E)** disease-specific survival; **(F)** without radiation therapy; and **(G)** with radiation therapy. **(H)** Protein expression of C1QA, C1QB, and C1QC in cutaneous melanoma from The Human Protein Atlas. **(I,J)** Prognostic significance of C1QA, C1QB, and C1QC in SKCM from GSE65904 and GSE53118: overall survival. **(K)** Relative mRNA expression of C1QA, C1QB, and C1QC in HaCaT, A375, and MV3 cells *via* RT-qPCR analysis (*p < 0.05, **p < 0.01, and ***p < 0.001).

To further explore the correlation between the expression of C1QA, C1QB, and C1Q and the survival prognosis of cancer patients, we conducted a survival analysis of different tumour types using TIMER based on the aforementioned GEPIA analysis results. Interestingly, we found a strong association between the expression of these three genes and OS in SKCM (Table 2). Based on the aforementioned findings, we concentrated on the involvement of C1QA, C1QB, and C1QC in SKCM. Box plots of expression differences and OS curves obtained from the GEPIA database showed that the overexpression of C1QA, C1QB, and C1QC prolonged the OS of SKCM patients (Figures 3A, B).

**TABLE 2:** Correlation between C1QA, C1QB, and C1QC and overall survival in cancers with significant differences in expression.

Cancer	Variable	*p-*value	Cancer	Variable	*p-*value	Cancer	Variable	*p-*value
SKCM	C1QA	1.62E–06	SKCM	C1QB	4.18E–06	SKCM	C1QC	2.31E–06
LGG	C1QA	0.002679	LGG	C1QB	0.00029	LGG	C1QC	0.000777
THYM	C1QA	0.006067	ESCA	C1QB	0.072503	THYM	C1QC	0.009601
KIRC	C1QA	0.044099	THYM	C1QB	0.113741	TGCT	C1QC	0.072349
ESCA	C1QA	0.058511	PAAD	C1QB	0.15336	ESCA	C1QC	0.078537
PAAD	C1QA	0.129492	OV	C1QB	0.159892	PAAD	C1QC	0.127006
TGCT	C1QA	0.183879	KIRC	C1QB	0.162042	KIRC	C1QC	0.311248
GBM	C1QA	0.22455	TGCT	C1QB	0.183879	UCEC	C1QC	0.379736
STAD	C1QA	0.344496	LUSC	C1QB	0.30921	STAD	C1QC	0.444084
KIRP	C1QA	0.369394	KIRP	C1QB	0.310142	HNSC	C1QC	0.488506
UCEC	C1QA	0.440934	UCEC	C1QB	0.409936	ACC	C1QC	0.49011
LIHC	C1QA	0.536671	GBM	C1QB	0.423744	KIRP	C1QC	0.533241
LUSC	C1QA	0.539205	DLBC	C1QB	0.508697	OV	C1QC	0.534026
OV	C1QA	0.58539	STAD	C1QB	0.515331	DLBC	C1QC	0.573832
DLBC	C1QA	0.59649	HNSC	C1QB	0.633914	GBM	C1QC	0.714599
UCS	C1QA	0.878045	ACC	C1QB	0.814952	UCS	C1QC	0.731257
			LUAD	C1QB	0.955506			

The SKCM dataset was downloaded from TCGA to investigate the association between the expression of these three genes and OS, progression-free interval (PFI), and disease-specific survival (DSS). The results revealed that the overexpression of C1QA, C1QB, and C1QC had a significantly better prognostic value for OS, PFI, and DSS (Figure 3C–E). In addition, high expression of these three genes in SKCM improved OS for patients regardless of whether they received radiation therapy. These results suggested that radiotherapy did not affect the prognostic effects of these three genes in SKCM patients (Figures 3F, G). The analysis results of the HPA database also showed that the expression of proteins encoded by C1QA and C1QC was significantly increased in SKCM compared with that in normal skin tissue, but the protein encoded by C1QB was not detected in either tissue (Figure 3H). In addition, the association of C1QA, C1QB, and C1QC with OS was studied using the GEO database (GSE65904 and GSE53118). In terms of OS, the analysis results of C1QB and C1QC were consistent with those of TCGA data, and a high expression predicted a better prognosis. Although C1QA did not correlate well with overall survival, a high expression was associated with a better prognosis (Figures 3I, J). Thus, we can conclude that the overexpression levels of C1QA, C1QB, and C1QC are protective factors which improve survival in SKCM patients at different stages. Furthermore, we detected the expression levels of three genes in A375 and MV3 melanoma cells and normal HaCaT cells using RT-qPCR. As shown in Figure 3K, the expression levels of the three genes were significantly increased in A375 and MV3 cells compared to those in HaCaT cells. These results suggest that these three genes are upregulated in cutaneous melanoma cells.

### C1QA, C1QB, and C1QC were independent prognostic factors in skin cutaneous melanoma patients

**FIGURE 4:**
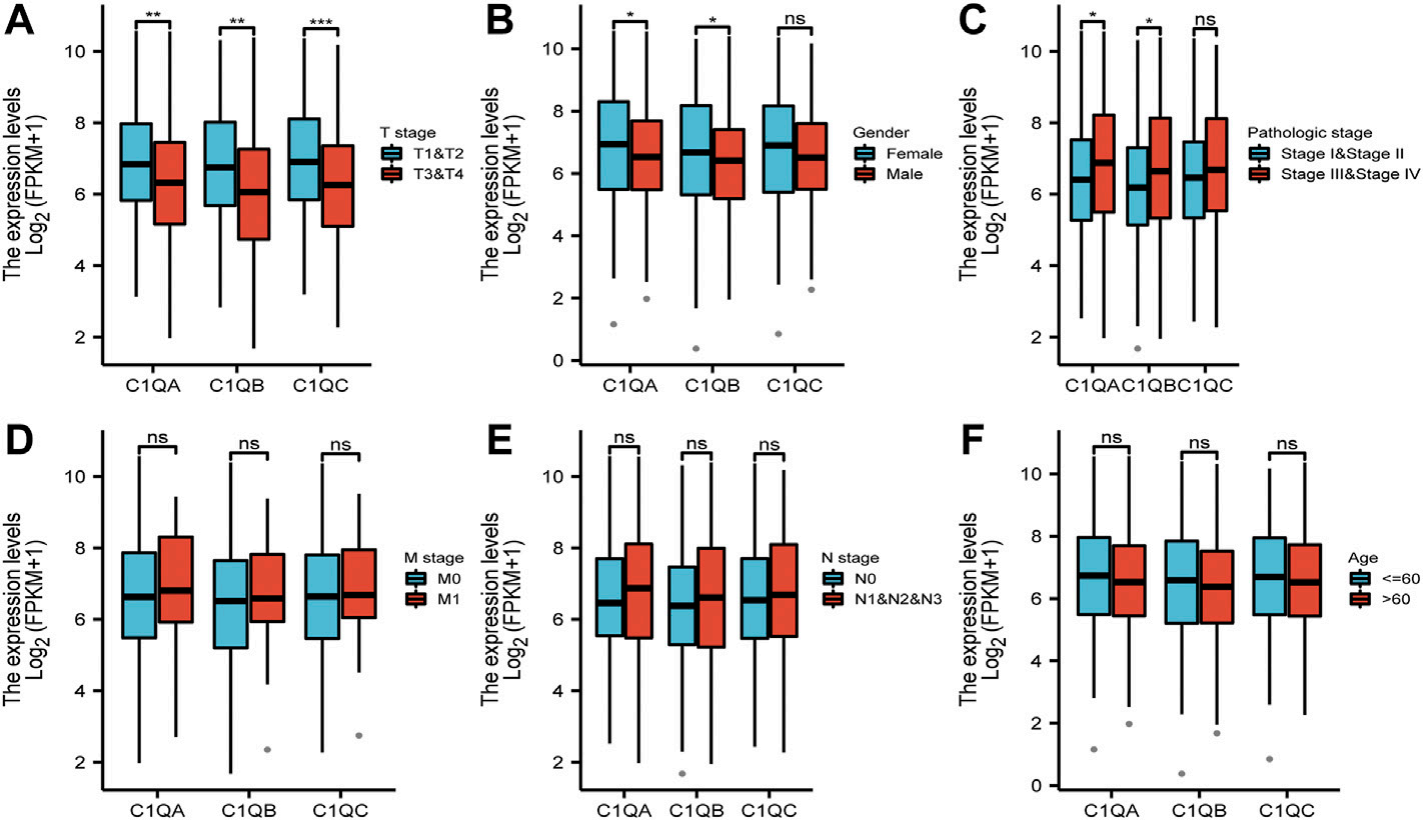
Correlation between differential expression of C1QA, C1QB, and C1QC and clinicopathological features: **(A)** T stage; **(B)** sex; **(C)** pathological stage; **(D)** M stage; **(E)** N stage; and **(F)** age (ns = no significance; *p < 0.05; **p < 0.01; ***p < 0.001).

Univariate and multivariate Cox regression analyses were performed on C1QA, C1QB, and C1QC with clinical variables to determine whether C1QA, C1QB, and C1QC could be distinguished from other clinical features as independent prognostic factors in SKCM patients. In conclusion, C1QA, C1QB, and C1QC can be distinguished from age and sex as independent prognostic factors for predicting patient survival, and the TNM stage can also be used as an independent prognostic factor for SKCM (Tables 3–5). In the aforementioned three tables, although radiotherapy is a protective factor for SKCM (Hazard ratio <1), its *p*-value is greater than 0.05, indicating that radiotherapy does not significantly affect the prognosis of melanoma patients. Based on the TCGA-SKCM dataset, we further explored the correlation between the expression of these three genes and their clinicopathological features. As shown in Figure 4, the expression of C1QA and C1QB was significantly correlated with pathological stage and sex, and the expression of all three genes was significantly correlated with T stage but not with M stage, N stage, and age.

**TABLE 3:** Univariate and multivariate Cox regression analyses of C1QA expression and clinicopathological characteristics with OS in SKCM patients.

Characteristic	Total (n)	Univariate analysis		Multivariate analysis	
		Hazard ratio (95% CI)	*p*-value	Hazard ratio (95% CI)	*p*-value
T stage	361				
T1 and T2	118	Reference			
T3 and T4	243	2.085 (1.501–2.895)	**<0.001**	1.807 (1.267–2.575)	**0.001**
N stage	402				
N0	224	Reference			
N1 and N2 and N3	178	1.752 (1.304–2.354)	**<0.001**	2.821 (0.991–8.033)	0.052
M stage	430				
M0	406	Reference			
M1	24	1.897 (1.029–3.496)	**0.040**	2.744 (1.138–6.617)	**0.025**
Pathological stage	410				
Stage I and Stage II	217	Reference			
Stage III and Stage IV	193	1.617 (1.207–2.165)	**0.001**	0.660 (0.228–1.910)	0.443
Gender	456				
Female	172	Reference			
Male	284	1.172 (0.879–1.563)	0.281		
Age	456				
<=60	246	Reference			
>60	210	1.656 (1.251–2.192)	<0.001	1.282 (0.918–1.789)	0.144
radiation therapy	450				
no	374	reference			
yes	76	0.977 (0.694–1.377)	0.895		
C1QA	456				
Low	227	Reference			
High	229	0.540 (0.411–0.708)	**<0.001**	0.561 (0.405–0.776)	**<0.001**

Abbreviation: CI, confidence interval.

Bold values: *p* < 0.05.

**TABLE 4:** Univariate and multivariate Cox regression analyses of C1QB expression and clinicopathological characteristics with OS in SKCM patients.

Characteristic	Total (n)	Univariate analysis		Multivariate analysis	
		Hazard ratio (95% CI)	*p*-value	Hazard ratio (95% CI)	*p*-value
T stage	361				
T1 and T2	118	Reference			
T3 and T4	243	2.085 (1.501–2.895)	**<0.001**	1.859 (1.305–2.648)	**<0.001**
N stage	402				
N0	224	Reference			
N1 and N2 and N3	178	1.752 (1.304–2.354)	**<0.001**	2.605 (0.922–7.362)	0.071
M stage	430				
M0	406	Reference			
M1	24	1.897 (1.029–3.496)	**0.040**	2.208 (0.927–5.257)	0.074
Pathological stage	410				
Stage I and Stage II	217	Reference			
Stage III and Stage IV	193	1.617 (1.207–2.165)	**0.001**	0.695 (0.243–1.990)	0.498
Sex	456				
Female	172	Reference			
Male	284	1.172 (0.879–1.563)	0.281		
Age	456				
<=60	246	Reference			
>60	210	1.656 (1.251–2.192)	<0.001	1.257 (0.901–1.753)	0.178
radiation therapy	450				
no	374	reference			
yes	76	0.977 (0.694–1.377)	0.895		
C1QB	456				
Low	229	Reference			
High	227	0.578 (0.441–0.758)	**<0.001**	0.593 (0.429–0.820)	**0.002**

Abbreviation: CI, confidence interval.

Bold values: *p* < 0.05.

**TABLE 5:** Univariate and multivariate Cox regression analysis of C1QC expression and clinicopathological characteristics with OS in SKCM patients.

Characteristic	Total (n)	Univariate analysis		Multivariate analysis	
		Hazard ratio (95% CI)	*p*-value	Hazard ratio (95% CI)	*p*-value
T stage	361				
T1 and T2	118	Reference			
T3 and T4	243	2.085 (1.501–2.895)	**<0.001**	1.839 (1.291–2.621)	**<0.001**
N stage	402				
N0	224	Reference			
N1 and N2 and N3	178	1.752 (1.304–2.354)	**<0.001**	2.894 (1.023–8.192)	**0.045**
M stage	430				
M0	406	Reference			
M1	24	1.897 (1.029–3.496)	**0.040**	2.509 (1.051–5.988)	**0.038**
Pathological stage	410				
Stage I and Stage II	217	Reference			
Stage III and Stage IV	193	1.617 (1.207–2.165)	**0.001**	0.645 (0.225–1.854)	0.416
Sex	456				
Female	172	Reference			
Male	284	1.172 (0.879–1.563)	0.281		
Age	456				
<=60	246	Reference			
>60	210	1.656 (1.251–2.192)	<0.001	1.267 (0.908–1.769)	0.164
radiation therapy	450				
no	374	reference			
yes	76	0.977 (0.694–1.377)	0.895		
C1QC	456				
Low	226	Reference			
High	230	0.569 (0.434–0.745)	**<0.001**	0.607 (0.440–0.837)	**0.002**

Abbreviation: CI, confidence interval.

Bold values: *p* < 0.05.

### Diagnostic and predictive values of C1QA, C1QB, and C1QC

**FIGURE 5:**
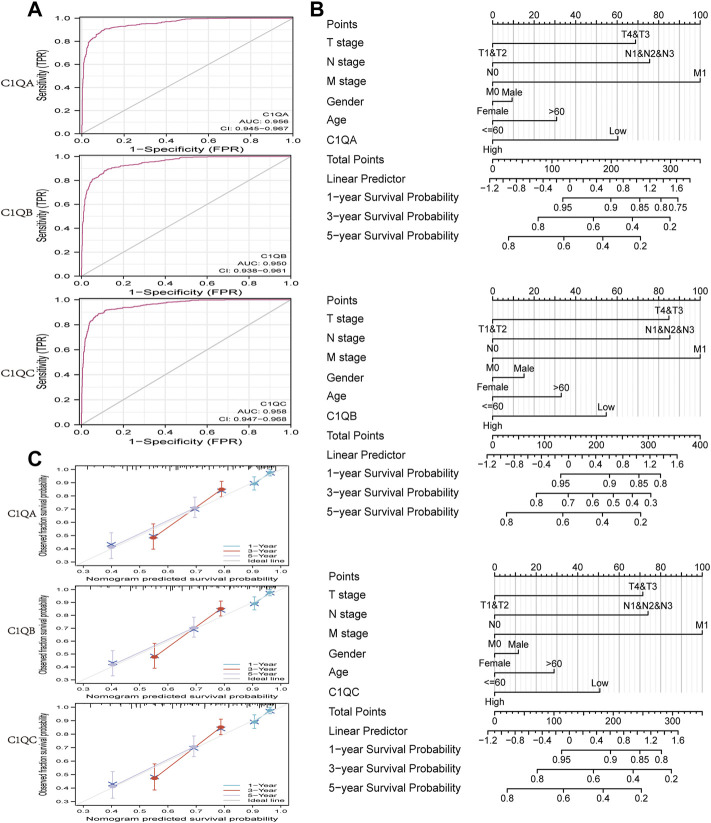
ROC curve and nomogram. **(A)** ROC curve in normal tissue and cutaneous melanoma. **(B)** Nomogram for predicting the probability of SKCM patients with 1-, 3-, and 5-year OS. **(C)** Calibration curves of the nomogram between predicted and actual 1-, 2-, and 3-year OS.

The area under the ROC curve (AUC) was used to evaluate the diagnostic performance of C1QA, C1QB, and C1QC. As shown in Figure 5A, the areas under the curve (AUC) of C1QA, C1QB, and C1QC were 0.956 (CI: 0.945–0.967), 0.950 (CI: 0.938–0.961), and 0.958 (CI: 0.947–0.968), respectively. These findings suggest that all three genes are promising diagnostic markers for predicting SKCM, with C1QC being the best predictor, followed by C1QA and C1QC. We then visualised the results of the multivariate Cox proportional risk analysis as a nomogram to predict 1-, 3-, and 5-year OS (Figure 5B). Furthermore, the C-indexes of C1QA, C1QB, and C1QC were 0.687 (95% CI: 0.665–0.710), 0.684 (95% CI: 0.662–0.706), and 0.684 (95% CI: 0.662–0.707), respectively. Calibration further confirmed the accuracy of the nomogram’s prediction ability. As shown in Figure 5C, the calibration curves showed a good fit to the diagonal, indicating that the predicted values were generally consistent with the actual observed values of the 1-, 3-, and 5-year OS probabilities.

### Differentially expressed genes related to C1QA, C1QB, and C1QC in skin cutaneous melanoma

**FIGURE 6:**
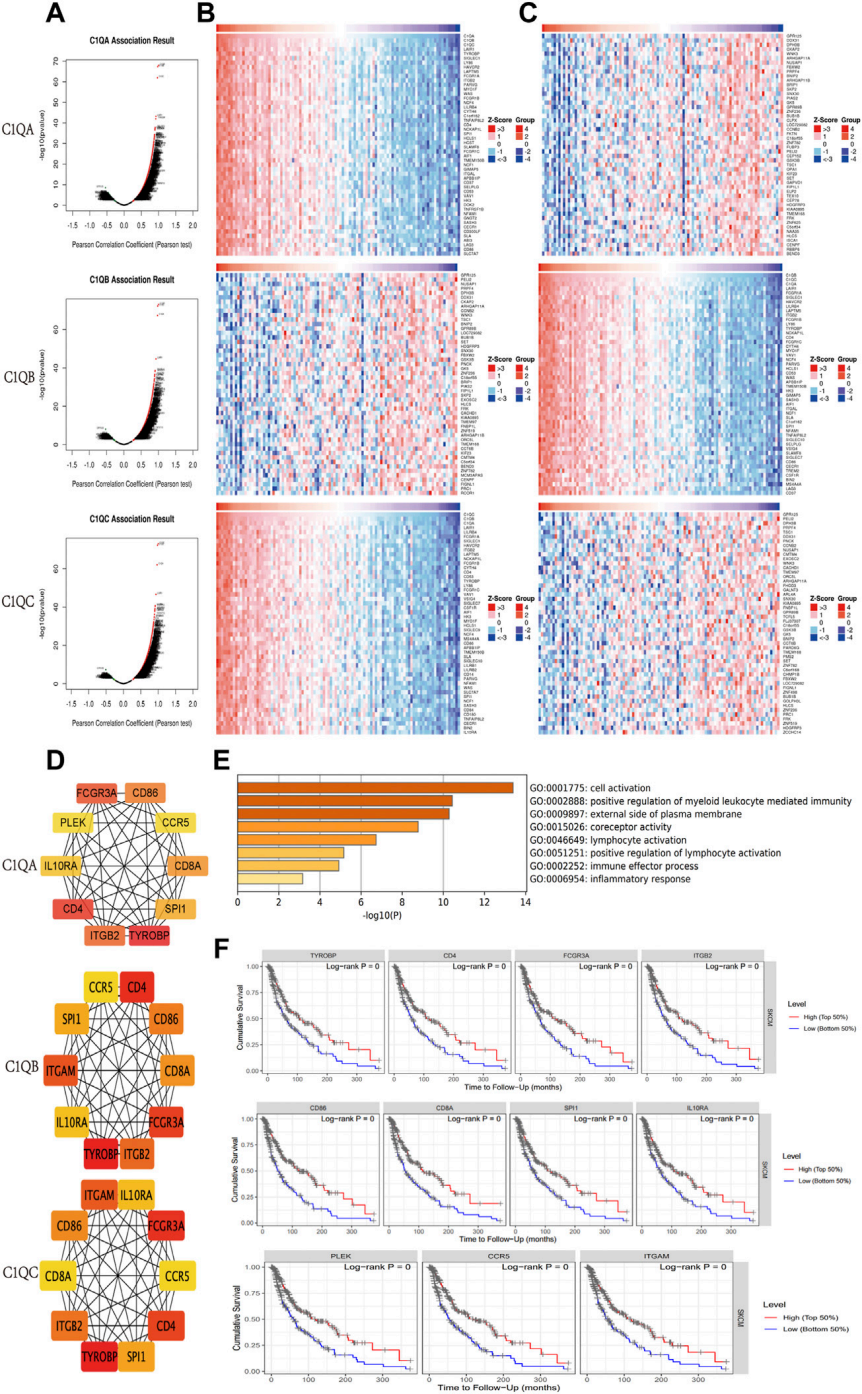
Genes correlated with C1QA, C1QB, and C1QC in SKCM. **(A)** Correlations between C1QA, C1QB, and C1QC and differently expressed genes. **(B,C)** Top 50 positively or negatively correlated genes. **(D)** Top 10 hub genes. **(E)** GO analysis of 11 hub genes. **(F)** Effect of 11 hub genes on OS in patients with SKCM.

Using the LinkedOmics online program, we identified DEGs that interacted with C1QA, C1QB, and C1QC in patients with SKCM. The volcano plot showed that most DEGs were positively correlated with C1QA, C1QB, and C1QC (Figure 6A). The top 50 genes that were positively or negatively associated with C1QA, C1QB, and C1QC is shown in the heat map (Figures 6B, C). Based on a correlation greater than 0.7, we uploaded 214 C1QA-related DEGs, 213 C1QB-related DEGs, and 202 C1QC-related DEGs to the STRING database to analyse the interactions between the proteins they encode. The ten hub genes with the most interactions in each group were obtained using the Cytoscape tool (Figure 6D). We identified 30 hub genes from the three groups. After removing duplicate genes, 11 hub genes were used for subsequent analyses. Function and pathway enrichment analyses of these 11 hub genes were performed using the Metascape website. As shown in Figure 6E, hub genes were mainly involved in immune system-related biological processes, such as positive regulation of myeloid leukocyte-mediated immunity, external side of the plasma membrane, lymphocyte activation, positive regulation of lymphocyte activation, immune effector process, and immune receptor activity. In addition, all hub genes were found to have a good prognosis in SKCM using TIMER (Figure 6F). Based on these data, we inferred that the relationship between central genes may contribute to C1QA, C1QB, and C1QC, thus prolonging survival in patients with cutaneous melanoma.

### Positive correlation with immune cell infiltration

**FIGURE 7:**
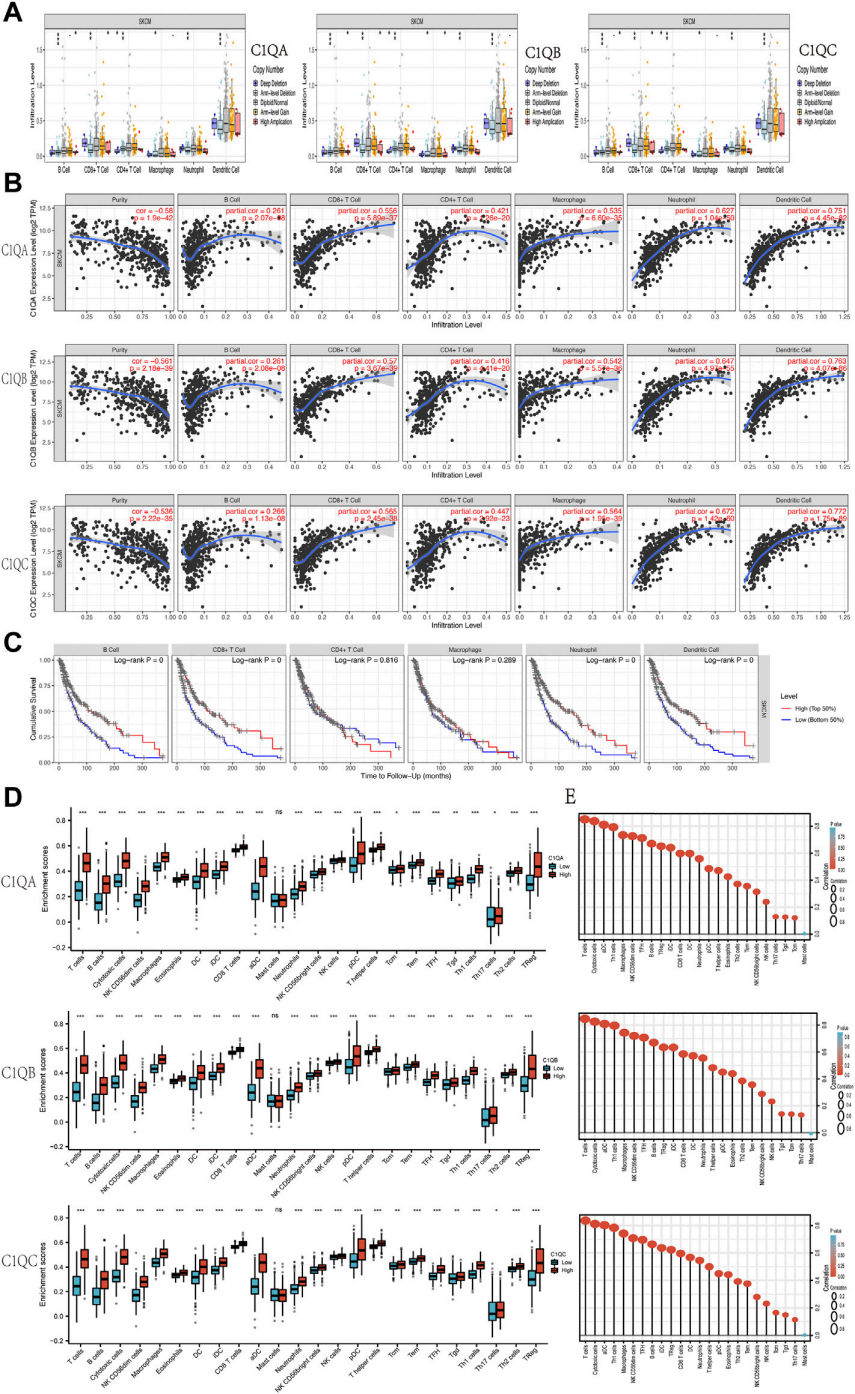
Correlation analysis of C1QA, C1QB, and C1QC expression with tumour-infiltering immune cells in SKCM. **(A)** Relationships between infiltration levels of six immune cells and copy number of C1QA, C1QB, and C1QC. **(B)** Relationships between infiltration levels of six immune cells and C1QA, C1QB, and C1QC expression. **(C)** Relationships between infiltration levels of six immune cells and SKCM prognosis. **(D)** Difference of infiltration levels of 24 immune cells between C1QA, C1QB, and C1QC high-expression and low-expression groups. **(E)** Correlation between the relative abundances of 24 immune cells and C1QA, C1QB, and C1QC expression levels (ns = no significance; *p < 0.05; **p < 0.01; ***p < 0.001).

C1Q is a member of the complement system and plays a role in a range of immunobiological processes (Kouser et al., 2015; Son et al., 2015). Therefore, we used TIMER to determine whether the expression of the three genes in SKCM was related to the level of immune cell infiltration. At various copy counts of C1QA, C1QB, and C1QC, we discovered substantial differences in immune cell infiltration (Figure 7A). We then investigated the relationship between the expression levels of these three genes and immune cell infiltration (Figure 7B). We concluded that the expression levels of C1QA, C1QB, and CIQC in SKCM were positively correlated with B cells, CD8^+^ and CD4^+^ T cells, macrophages, neutrophils, and dendritic cells. In addition, B cells, CD8^+^ T cells, neutrophils, and dendritic cells were defence factors associated with the cumulative survival over time in SKCM (Figure 7C).

These findings suggest that high expression levels of C1QA, C1QB, and C1QC are significantly associated with immune cell infiltration in patients with melanoma and may contribute to improved patient outcomes. ssGSEA was used to further verify whether the expression of the three genes affected the infiltration of the 24 immune cell subsets in SKCM. As shown in Figures 7D, E, the expression of C1QA, C1QB, and C1QC was strongly associated with 23 types of immune cells. Based on these results, we can speculate that C1QA, C1QB, and C1QC play key roles in immune cell infiltration in the tumour microenvironment of SKCM.

### C1QA, C1QB, and C1QC were positively correlated with immune cell markers

To further understand the relationship between C1QA, C1QB, and C1QC, and various immune infiltrating cells, we used GEPIA to analyse the correlation between these three genes and various immune markers in SKCM. As shown in Table 6, in SKCM, C1QA, C1QB, and C1QC were all positively correlated with immune markers, such as B cells (CD19 and CD79A), CD8^+^ T cells (CD8A and CD8B), CD4^+^ T cells (CD4), M1 macrophages (IRF5), M2 macrophages (CD163, VSIG4, and MS4A4A), immune markers of neutrophils (ITGAM and CCR7), and immune markers of dendritic cells (HLA-DPB1, HLA-DQB1, HLA-DRA, HLA-DPA1, CD1C, NRP1, and ITGAX).

**TABLE 6:** Correlation analysis between C1QA, C1QB, and C1QC and immune cell markers in SKCM using GEPIA.

Immune cell	Biomarker	C1QA R	*p*	C1QB R	*p*	C1QC R	*p*
B cell	CD19	0.58	***	0.58	***	0.57	***
	CD79A	0.64	***	0.63	***	0.61	***
CD8^+^ T cell	CD8A	0.86	***	0.86	***	0.84	***
	CD8B	0.84	***	0.83	***	0.81	***
CD4^+^ T cell	CD4	0.88	***	0.89	***	0.89	***
M1 macrophage	INOS (NOS2)	−0.018	0.71	−0.012	0.79	0.0071	0.88
	IRF5	0.67	***	0.67	***	0.68	***
	COX2 (PTGS2)	−0.049	0.3	−0.048	0.3	−0.039	0.4
M2 macrophage	CD163	0.83	***	0.84	***	0.85	***
	VSIG4	0.84	***	0.86	***	0.86	***
	MS4A4A	0.86	***	0.87	***	0.89	***
Neutrophil Dendritic cell	CD66b (CEACAM8)	−0.021	0.65	−0.017	0.71	−0.014	0.76
	CD11b (ITGAM)	0.77	***	0.78	***	0.79	***
	CCR7	0.66	***	0.65	***	0.65	***
	HLA-DPB1	0.84	***	0.84	***	0.83	***
	HLA-DQB1	0.68	***	0.67	***	0.66	***
	HLA-DRA	0.85	***	0.85	***	0.84	***
	HLA-DPA1	0.82	***	0.82	***	0.82	***
	BDCA-1 (CD1C)	0.51	***	0.5	***	0.5	***
	BDCA-4 (NRP1)	0.35	***	0.37	***	0.4	***
	CD11c (ITGAX)	0.65	***	0.65	***	0.64	***

Cor, R-value of Spearman’s correlation; *p < 0.01; **p < 0.001; ***p < 0.0001.

### Positive correlation with immune checkpoints

**FIGURE 8:**
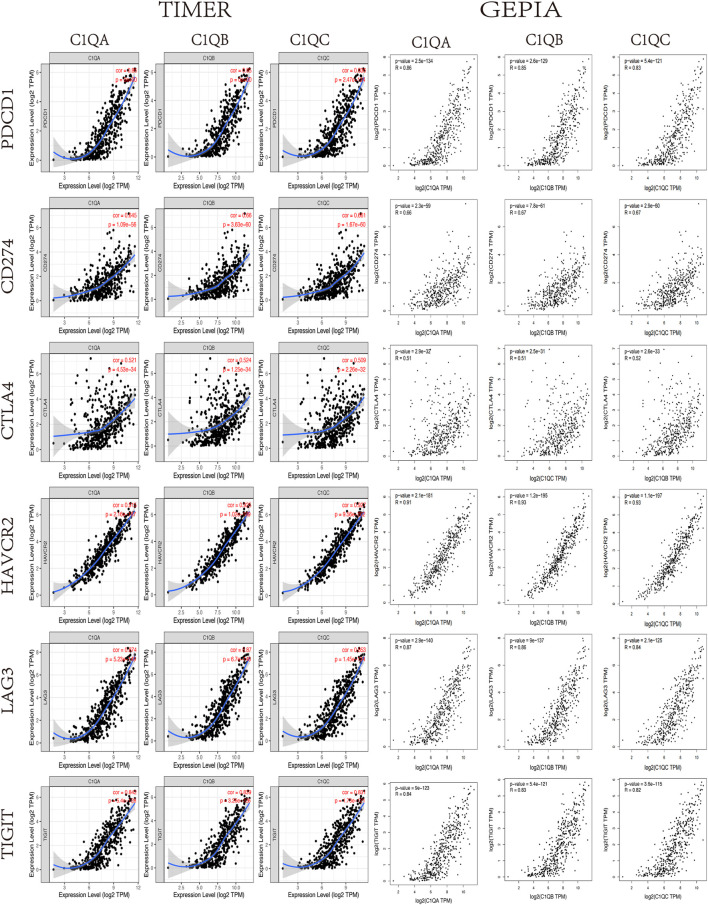
Correlation between C1QA, C1QB, and C1QC expression levels and immune checkpoints in SKCM *via* TIMER analysis and GEPIA.

It is well known that in the field of tumour immunotherapy, five immune checkpoints, namely, PD-1, CTLA-4, Tim-3, LAG-3, and TIGIT, have inhibitory effects on T-cell proliferation. Immunosuppressive molecules are expressed in many different types of tumours, and blocking these molecules boosts the activation of immune cells. Therefore, we assessed the relationship between C1QA, C1QB, and C1QC and the five immune checkpoints using TIMER. Figure 7 shows that PD-1 (PDCD1), PD-L1 (CD274), CTLA-4 (CTLA4), Tim-3 (HAVCR2), LAG-3 (LAG3), and TIGIT immunosuppressive molecules were significantly and positively correlated with C1QA, C1QB, and C1QC expression. Analysis of the GEPIA data also verified a substantial positive relationship between these three genes and immunosuppressive molecules in SKCM (Figure 8). These results suggest that judicious use of inhibitors can help enhance the anti-tumour immune response and thus provide actual benefits to patients.

### Gene set enrichment analysis

**FIGURE 9:**
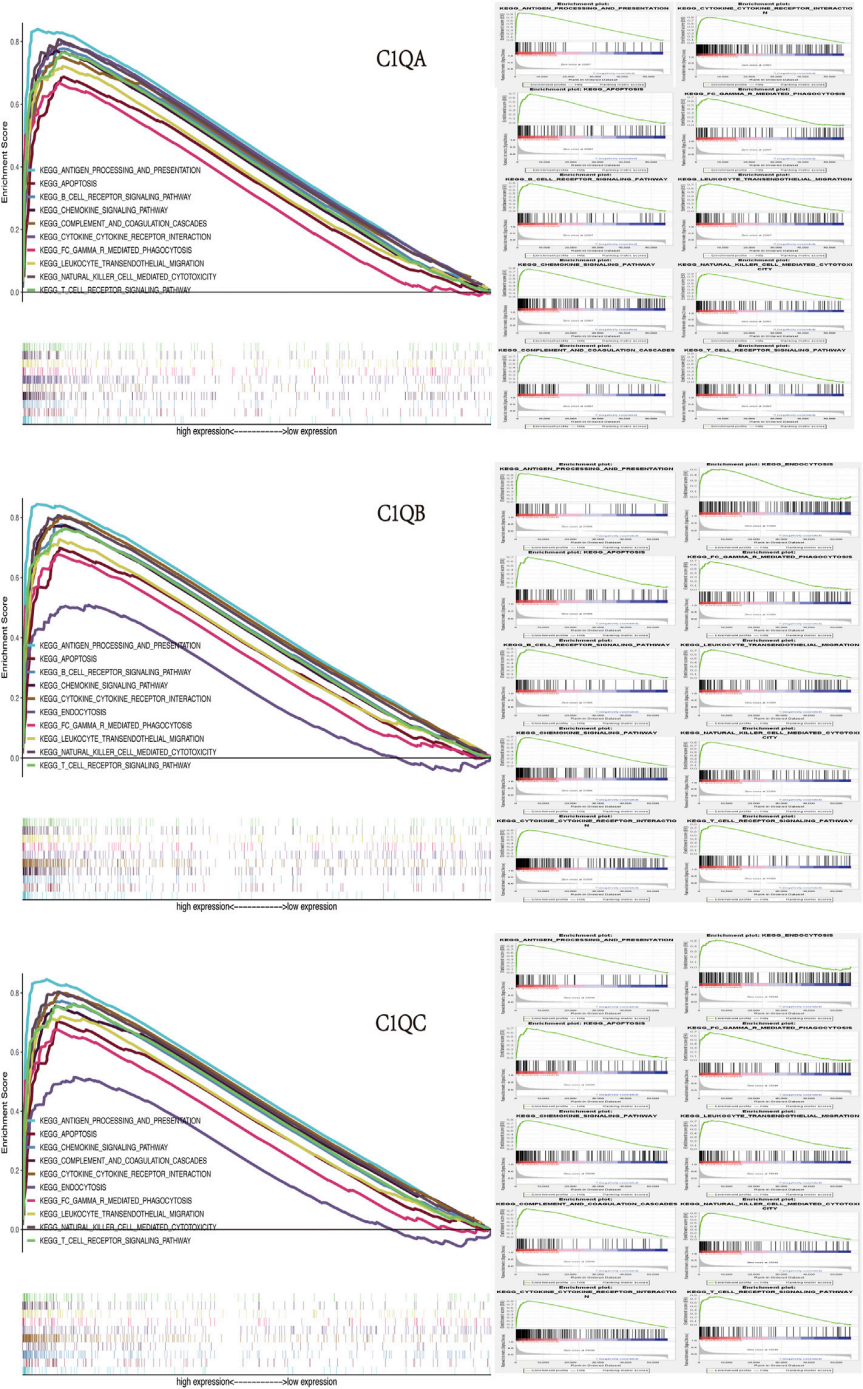
GSEA of C1QA, C1QB, and C1QC high-expression groups revealed enriched biological pathways in SKCM.

Finally, we identified the corresponding enrichment pathways in the high- and low-expression groups of C1QA, C1QB, and C1QC using GSEA. Surprisingly, the signalling pathways enriched by the three genes in the high-expression group were almost identical (Figure 9), including “antigen processing and presentation,” “B-cell receptor signalling pathway,” “T-cell receptor signalling pathway,” “natural killer cell-mediated cytotoxicity,” “leukocyte transendothelial migration,” “Fc gamma R-mediated phagocytosis,” “cytokine–cytokine receptor interaction,” and “chemokine signalling pathway.” In addition to their important roles in anti-tumour immunity, C1QA, C1QB, and C1QC were also significantly enriched in apoptotic pathways.

## Discussion

SKCM is the most life-threatening form of skin neoplasm, affecting millions of individuals worldwide each year (Corrie et al., 2014). SKCM often has a poor prognosis because of its high resistance to radiation and chemotherapeutic drugs (Pollack et al., 2011; Polkowska et al., 2016; Sandri et al., 2016). Elucidating the molecular mechanisms involved in the carcinogenesis of SKCM may contribute to the study of potential therapeutic targets and the development of valuable diagnostic and prognostic biomarkers. Complement is an important part of the innate immune system, which plays a role in resisting pathogen invasion and protecting the host (Roumenina et al., 2019a). Although C1Q has been proved to promote cell death in most types of cancers, in some cancers, the role of C1Q is completely opposite, which leads to cancer progression (Revel et al., 2022). In clear-cell renal cell carcinoma, tumour-associated macrophages generate C1r, C1s, C4, and C3, after being hijacked by cancer cells, and then initiates the classical pathway of the complement cascade of IgG immunity in tumour and promotes inflammation and T-cell exhaustion, leading to tumour progression (Roumenina et al., 2019b). Also, a high expression of C1Q is positively correlated with a poor prognosis in glioma of different grades (Mangogna et al., 2019a). In ovarian cancer, C1Q induced apoptosis in the SKOV3 cell line *via* the TNF-α-induced apoptosis pathway up-regulated by Bax and Fas, confirming the potential protective effect of C1Q Kaur et al. (2016). It is worth mentioning that C1Q-deficient mice carrying homologous B16 melanomas had slower tumour growth and longer survival than wild-type or C3- or C5-deficient mice. This study confirmed the role of C1Q in promoting melanoma growth. However, there are few reports about the specific role of C1Q in melanoma patients (Bulla et al., 2016). In recent years, with the rapid development of bioinformatics, gene exploration based on public databases has been widely used in medical research and plays an active role in clinical diagnosis, treatment, and prognosis prediction. Based on the existing public database and the validation of basic experiments, this study deeply explored the role of C1Q expression in diagnosis, prognosis and tumour immunity of SKCM patients, aiming to provide a reference for further research on the specific mechanism of C1Q in the future.

In the present study, we used the GEPIA database to conduct pan-cancer analysis on the transcription levels of C1QA, C1QB, and C1QC and screened out the cancer types with the differential expression of these three genes. We then performed a survival analysis of these differentially expressed cancers using the TIMER database and found that in SKCM, the expression levels of C1QA, C1QB, and C1QC were significantly correlated with OS. Based on this conclusion, we performed a more comprehensive survival analysis of C1QA, C1QB, and C1QC using expression data and clinical data from TCGA-SKCM. The results showed that the high expression of C1QA, C1QB, and C1QC in SKCM patients predicted a better prognosis and was not affected by radiation therapy. To ensure that the results were more reliable, we also analysed and verified OS and differential expression using GEO and HPA databases, verified differential expression using RT-qPCR, and reached a consistent conclusion. In addition, the current study found significant differences in the expression of C1QA, C1QB, and C1QC in patients with different T and pathological stages; the earlier these stages, the higher the expression of C1QA, C1QB, and C1QC and the better the prognosis. Therefore, these complement components are considered to play a role in inhibiting tumour development to a certain extent. Finally, multivariate Cox regression analysis and ROC analysis confirmed that C1QA, C1QB, and C1QC can be used as independent prognostic biomarkers and contribute to the diagnosis of SKCM.

The nomogram scale has been widely used to predict the OS of cancer patients. Based on C1QA, C1QB, C1QC, and six clinical parameters (sex, age, pathological stage, T stage, N stage, and M stage), we created individual nomograms to predict the 1-, 3-, and 5-year OS of SKCM patients and verified the accuracy of nomograms by the C-index and calibration curve. In addition, among the 11 identified C1QA/C1QB/C1QB-related hub genes, we found that their overall function was mapped to immune-related activity, and the high expression of these hub genes predicted better OS. These results suggest that these hub genes may have a synergistic effect on C1QA, C1QB, and C1QC and improve the prognosis of SKCM patients through immune response function.

In recent years, the role of the immune system in cancer development has been studied extensively. Immunotherapy, including vaccines, adoptive cell transfer, and immune detection blockers, has rapidly developed as a promising therapeutic strategy for malignant tumours. Previous studies have reported that tumour immune cell infiltration can improve the prognosis of SKCM and efficacy of immunotherapy (Fu et al., 2019; Selitsky et al., 2019). Adoptive cell therapy with autologous tumour-infiltrating lymphocytes is an effective treatment for metastatic SKCM (Rosenberg et al., 2011). To further understand the mechanism of C1QA, C1QB, and C1QC in SKCM carcinogenesis, we explored the relationship between C1QA, C1QB, and C1QC and immune cell infiltration. Our results showed that the expression levels of C1QA, C1QB, and C1QC in SKCM positively correlated with B cells, CD8^+^ and CD4^+^ T cells, macrophages, neutrophils, dendritic cells, and their biomarkers. Among these, B cells, CD8^+^ T cells, neutrophils, and dendritic cells play an active role in prolonging the OS of SKCM patients. Evidence confirms that tumour-infiltrating B cells play a key role in the anti-tumour immune response of SKCM (DiLillo et al., 2010; Gilbert et al., 2011). CD8^+^ T cells are an important component of the tumour immune response and exert cytotoxic effects on melanoma cells (Durgeau et al., 2018). Dendritic cell tumour vaccines are the earliest and most effective methods for the clinical treatment of melanoma (Gao et al., 2016). Furthermore, the neutrophil-to-lymphocyte ratio can be used as a biomarker for metastatic SKCM (Sellers et al., 2019). Finally, ssGSEA further confirmed the high correlation between C1QA, C1QB, and C1QC and immune cell infiltration. Based on the aforementioned results, it can be concluded that C1QA, C1QB, and C1QC may be protective factors and prolong the OS of SKCM patients by mediating immune cell infiltration.

It is worth mentioning that immunotherapy can produce an effective anti-tumour immune response that requires both enough immune cells to infiltrate the tumour microenvironment and the full expression of immune checkpoints (Chae et al., 2018). Recently, immune checkpoint inhibitors (ICIs), including ipilimumab (a CTLA-4 inhibitor) and nivolumab (a PD-1 inhibitor), have shown remarkable therapeutic effects on SKCM (Eggermont et al., 2018). Some studies have reported that PD-L1 in SKCM cells plays a key role by downregulating the function of T cells after binding to PD-1 on lymphocytes (Hamid et al., 2013). However, the main drawback of these ICIs is their low response efficiencies (Burtness et al., 2019). Therefore, patients with high PD-L1 expression tend to have better treatment outcomes than patients with low PD-L1 expression (Daud et al., 2016; Weide et al., 2016). The infiltration of massive CD8^+^ T cells within melanoma cells can enhance the clinical benefits of ICIs (Tumeh et al., 2014; Linette and Carreno, 2019; Wong et al., 2019). More importantly, the combination of anti-CTLA-4 and anti-PD-1/PD-L1 showed stronger anti-tumour activity than monotherapy (Ott et al., 2013; Wolchok et al., 2013; Larkin et al., 2015). In our analysis of immune-related functions, the expression levels of C1QA, C1QB, and C1QC were positively correlated not only with PD1, PD-L1, and CTLA-4 but also with the molecules of multiple immune checkpoints. This suggests that SKCM patients with high C1QA, C1QB, and C1QC expression levels can benefit more from immunotherapy. In addition, we analysed the signalling pathways in which C1QA, C1QB, and C1QC are involved in SKCM through GSEA. These findings indicated that signalling pathways related to immune function were significantly enriched in patients with high expression of C1QA, C1QB, and C1QC. Moreover, these three genes have been found to be involved in apoptosis. Previous reports have confirmed that C1Q can lead to apoptosis in ovarian, prostate, and breast cancer (Hong et al., 2009; Bandini et al., 2016; Kaur et al., 2016). Therefore, we speculated that the high expression of C1QA, C1QB, and C1QC may play a dual regulatory role through anti-tumour immunity and the induction of cancer cell apoptosis.

In conclusion, the current study showed that C1QA, C1QB, and C1QC were highly expressed in a variety of cancers, especially in SKCM, which overexpressed these three complement components and predicted better survival prognosis. In addition, C1QA, C1QB, and C1QC increased immune cell infiltration and immune checkpoint expression in SKCM, helping patients benefit more from immunotherapy. However, the present study has some limitations. Our analysis and validation are mainly based on bioinformatics and melanoma cell lines. Therefore, in future studies, we need to perform *in vivo* experiments to confirm our outcomes.

## Data Availability

The original contributions presented in the study are included in the article/Supplementary Material; further inquiries can be directed to the corresponding author.
